# Water Residues from Rosemary Essential Oil Production: Transforming Waste into a Potential Bioherbicide

**DOI:** 10.3390/plants14172717

**Published:** 2025-09-01

**Authors:** Federico Leoni, Francesco Nigro, Celia Duce, José González-Rivera, Marco Mattonai, Erika Ribechini, Paolo Bàrberi, Stefano Carlesi

**Affiliations:** 1 Group of Agroecology, Institute of Plant Sciences, Scuola Superiore Sant’Anna, Piazza Martiri della Libertà, 33, 56127 Pisa, Italy; federico.leoni@santannapisa.it (F.L.); francesco.nigro@santannapisa.it (F.N.); stefano.carlesi@santannapisa.it (S.C.); 2 National Institute of Optics, (INO-CNR)—UOS Pisa, Via G. Moruzzi 1, 56124 Pisa, Italy; jose.gonzalezrivera@ino.cnr.it (J.G.-R.); 3 Department of Chemistry and Industrial Chemistry, University of Pisa, Via G. Moruzzi 13, 56124 Pisa, Italy; celia.duce@unipi.it (C.D.); marco.mattonai@unipi.it (M.M.); erika.ribechini@unipi.it (E.R.)

**Keywords:** natural herbicides, allelopathy, weed control, aromatic plant, medicinal plant, phenols, terpenes

## Abstract

Transforming industrial by-products into new resources is a fundamental principle of sustainable agriculture and circular bioeconomy. Waste products from rosemary (*Rosmarinus officinalis* L.) essential oil extraction, such as exhausted biomass and water residues (WRs), are rich in bioactive compounds like phenols and terpenes. These by-products may represent a promising and economically viable option for agricultural management, particularly in weed control. This study evaluates the potential use of WR as a bioherbicide. In vitro experiments were conducted to assess the inhibitory effects of WR on the germination and seedling morphology (root and shoot development) of four detrimental weed species for temperate cropping systems: two monocotyledonous (*Alopecurus myosuroides* and *Lolium multiflorum*) and two dicotyledonous (*Sinapis alba* and *Amaranthus retroflexus*). WR was tested at four concentrations (0, 25, 50, and 100), corresponding to an increasing gradient of WR, with 100 representing pure WR. The results showed that WR did not significantly inhibit germination in *A. myosuroides*, *L. multiflorum* and *S. alba*, whereas *A. retroflexus* exhibited a dose-dependent inhibition, with germination reduced by 37.5%, 64.5%, and 91.6% at doses of 25, 50, and 100, respectively, compared with the control (dose 0). Furthermore, germination delays were observed across all tested species with promising application of WR for regulating weed–crop competitive interactions in the early crop growth stages. Results on the morphological traits of weed seedlings showed that WR application affected root more than shoot growth inhibition. In particular, WR demonstrated a pronounced root inhibitory effect in *A. myosuroides*, *L. multiflorum*, and *A. retroflexus*. In contrast, a dose-dependent increase in root length was observed for *S. alba* (21.41 mm at dose 0 and 25.77 mm, 30.97 mm and 35.96 mm, respectively, at doses 25, 50, and 100). The results of this study highlight the potential application of WR as a sustainable solution to be included in an integrated weed management (IWM) toolbox and underscore their role in promoting the valorization of waste from essential oil production.

## 1. Introduction

Rosemary (*Rosmarinus officinalis* L.) is an aromatic and medicinal plant (AMP) widely distributed throughout the Mediterranean basin [1]. Its cultivation requires low inputs, making this crop a good candidate for valorizing marginal farmland typical of Mediterranean areas [2]. Rosemary is grown for diverse purposes, ranging from fresh biomass production for direct consumption to essential oil extraction. The essential oil yield from rosemary biomass is relatively low, ranging from 0.33% to 2.28% (*w*/*w*) of fresh biomass, and it is influenced by several factors, including cultivar choice, agronomic management, and environmental conditions [3,4,5,6,7]. Essential oils, commonly extracted through methods like hydro-distillation and steam distillation, are primarily used in high-value industries such as pharmacology, cosmetics, and food flavoring due to their costly and inefficient production process [8,9]. Essential oils also find application in agriculture, demonstrating their effectiveness as insect repellents, fungicides, and herbicides, with performance often comparable to that of synthetic products [10]. However, their widespread use in the agricultural sector is still limited due to low yields and expensive extraction processes, which often make large-scale applications economically unfeasible [11]. Instead, a more economically viable solution could be the use of by-products from essential oil extraction, which constitute the major component of the process output and have interesting chemical and physical properties, offering significant potential for valorization.

After the essential oil extraction process, two primary types of waste are generated: (i) exhausted biomass, consisting of solid residues from leaves, flowers, and stems, which accounts for 60–65% of the process output, and (ii) condensed water, known as water residues (WRs), which makes up 30–35% of the process output. While exhausted biomass, rich in lignocellulose, can be used for multiple agricultural applications, such as mulching, energy production through combustion, biochar conversion, bio-oil generation, or as a mushroom cultivation substrate [12,13,14,15], WR is usually discarded as wastewater, posing disposal challenges and environmental concerns.

This study explores the potential use of WR as a bioherbicide. Water residues (WRs) from rosemary extraction are particularly interesting due to their chemical composition and low cost. Many secondary metabolites with allelopathic potential are water-soluble during the extraction process, where steam distillation breaks part of the plant cellular walls, allowing these compounds to dissolve in the condensed water. As a result, WRs are enriched with a variety of water-soluble allelopathic substances, which are effective against weeds and may represent a sustainable alternative to synthetic herbicides or can contribute to reducing their use [1,16,17].

The WR chemical profile typically includes polyphenols, proteins/enzymes, amino acids, polysaccharides, alkaloids, alcoholic compounds, and vitamins, together with plant nutrients, such as Ca (38–755 mg/L), K (5–354 mg/L), Mg (0–52 mg/L), Na (6–31 mg/L), S (17–55 mg/L), and P (2–33 mg/L) [18,19]. Among polyphenolic compounds found in rosemary WR, there are phenolic diterpenes, such as carnosol, carnosic acid, rosmanol, epirosmanol, and isorosmanol, together with phenolic acids such as rosmarinic and caffeic acids. Notably, carnosic acid has been identified as the main contributor to the allelopathic activity of rosemary extracts, strongly inhibiting seedlings of lettuce growth in an in vitro experiment [20,21]. Similarly, rosmarinic acid has been shown to suppress root growth in *Arabidopsis thaliana* [22], while caffeic acid has demonstrated phytotoxic effects through oxidative stress induction and disruption of hormonal signaling in *Setaria viridis* and *Echinochloa crusgalli*, and the dicotyledonous weeds *Portulaca oleracea* and *Amaranthus retroflexus* [23]. However, the mode of action for many of the identified allelochemicals in WR is still unclear. Some mechanisms involve coating target seeds with hydrophobic compounds, making them impermeable to water and thereby hindering the activation of germination processes. Others interfere with the ability of roots to absorb water and nutrients, limiting their growth, as observed with sorgoleone extracted from sorghum [24]. In addition, several allelochemicals have been reported to interfere with the hormonal pathways regulating seed germination as well as hypocotyl and epicotyl development while also inducing oxidative stress that damages cellular structures and further constrains seedling establishment [23].

While synthetic herbicides are highly effective, their massive use has raised significant concerns about environmental pollution, human health, and herbicide resistance [25]. Instead, plant-derived bioherbicides, such as those from rosemary, might be well suited for sustainable agriculture due to their lower toxicity, persistence, and environmental impact. The use of such plant-based bioherbicides aligns with integrated weed management (IWM) strategies and sustainable agriculture principles.

Although bioherbicides account for less than 10% of all biopesticides globally [25], there is growing interest in research and the market for bioherbicides based on plant extracts and essential oils from medicinal and aromatic plants [26,27,28]. However, the use of these products in agriculture is still limited because they are very expensive to isolate and synthesize, regardless of having excellent herbicidal properties. In contrast, WRs are particularly appealing because they are a by-product of the essential oil extraction process rather than a substance that must be produced or extracted ex novo. By valorizing WR as a weed growth inhibitor, there is an opportunity to transform what would otherwise be waste into a valuable resource for agricultural use, addressing at once both economic and environmental concerns. In this study, WR was evaluated for its bioherbicidal potential by assessing its inhibitory effects on the germination and seedling morphology (root and shoot development) of four detrimental weed species affecting temperate cropping systems and with known problems related to herbicide resistance. We hypothesized the existence of a species-specific and targeted effect of WRs on different weed types. Therefore, in this experiment, we included two monocotyledon weed species (*Alopecurus myosuroides* and *Lolium multiflorum*) and two dicotyledons ones (*Sinapis alba* and *Amaranthus retroflexus*). To the best of our knowledge, studies on the use of WRs derived from essential oil extraction are very limited. This study aims to fill this gap by conducting an in vitro experiment to investigate the effect of WRs on seed germination and seedling morphology and to assess whether the effect is dose dependent.

## 2. Results

### 2.1. Weed Germination

**Figure 1:**
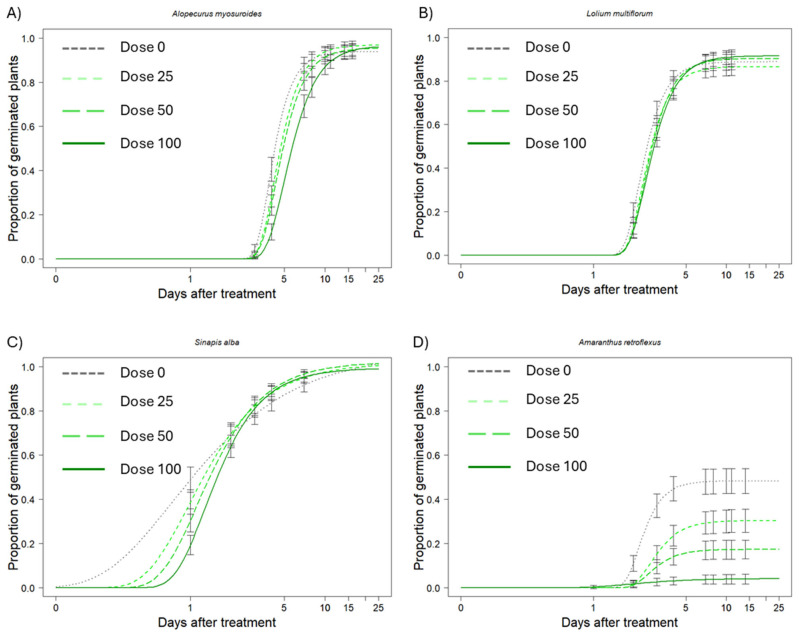
Germination dynamics of *Alopecurus myosuroides* (**A**), *Lolium multiflorum* (**B**), *Sinapis alba* (**C**), *Amaranthus retroflexus* (**D**) exposed to exhausted water residue (WR) at different concentrations (dose 0, dose 25, dose 50, dose 100). Values are means of five replicates, and DAT indicates day after treatment (log scale).

In the controls, i.e., without WR application, almost the totality of *A. myosuroides*, *L. multiflorum* and *S. alba* seeds were germinated (Table 1, Figure 1). *A. retroflexus*, however, had a maximum germination of 48%, confirming the high seed dormancy level in this species. Applying WR application at different concentrations did not show an inhibitory effect on the germination of *A. myosuroides*, *L. multiflorum*, and *S. alba*. In contrast, a clear inhibition effect was observed on *A. retroflexus*, with a significant decrease in germination as the WR concentration increased (Table 1, Figure 1). Specifically, germination inhibition was 37.5%, 64.5%, and 91.6%, respectively, for doses 25, 50, and 100 compared to the control (dose 0).

The situation appeared much more diversified when considering the slope and ET50 parameters. In general, the effect involved a delay in germination, which is reflected in higher absolute slope values in the control, which decreased with increasing WR concentration. The slope data were also confirmed by ET50 values, which showed a delay in reaching 50% germination in all the species examined. Specifically, for *A. myosuroides*, a significantly higher ET50 was observed at dose 50 compared to the control (Table 1, Figure 1). Meanwhile, for *L. multiflorum* and *S. alba*, a significant slowdown in the time required to reach ET50 was observed already at dose 25 (Table 1, Figure 1). Regarding *A. retroflexus*, an anomalous data point emerges: the ET50 at dose 100, which is not significantly different from the control (Table 1, Figure 1). These data should be interpreted with caution because only a few plants were germinated using dose 100, and they were not in sufficient number for correct statistical interpretation. This is also confirmed by the much higher standard error value compared to the rest of the data.

### 2.2. Root and Shoot Length of Weed Seedlings

**Figure 2:**
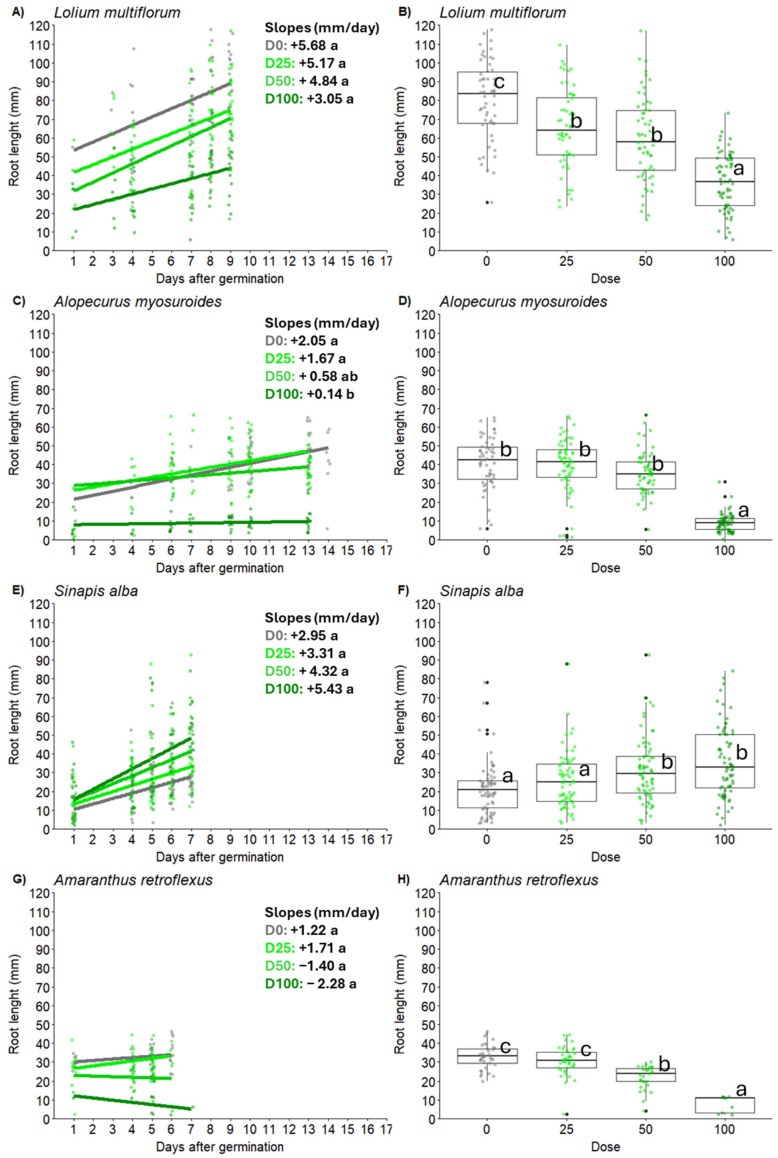
Root length dynamics (**left**) and mean root length (**right**) of seedlings of *Alopecurus myosuroides* (**A**,**B**), *Lolium multiflorum* (**C**,**D**), *Sinapis alba* (**E**,**F**), *Amaranthus retroflexus* (**G**,**H**), exposed to water residue (WR) at different concentrations (dose 0, dose 25, dose 50, dose 100). Values with the same letter are not significantly different at *p* ≤ 0.05.

Root length was significantly affected by WR application (*p* < 0.0001), weed species (*p* < 0.0001) and their interaction (*p* < 0.0001). Specific response effects changed according to the weed species and the WR concentration. For *A. myosuroides*, root length was found to be minimally influenced by WR at doses 25 and 50 but showed a significant reduction at dose 100 compared to the control (9.19 vs. 40.85 mm) (Figure 2). For *L. multiflorum*, a clear dose-dependent effect related to WR application was observed. A significant decrease in root length was observed already at dose 25 compared with the control (68.37 vs. 81.89 mm), reaching maximum growth inhibition at dose 100 (37.60 mm) (Figure 2). Similarly, for *A. retroflexus*, a dose-dependent effect was also observed, although less evident than in *L. multiflorum*. In this case, a significant reduction in root length compared to the control was observed at dose 50 compared with the control (19.92 vs. 32.46 mm), with minimum root length observed at dose 100 (7.28 mm) (Figure 2). Conversely, a contrasting trend was observed for *S. alba*, where root length increased with increasing WR dose application, following a dose-dependent trend. In this case, the root length of S. alba was 21.41 mm control and 25.77 mm, 30.97 mm and 35.96 mm, respectively, at doses 25, 50, and 100 (Figure 2).

**Figure 3:**
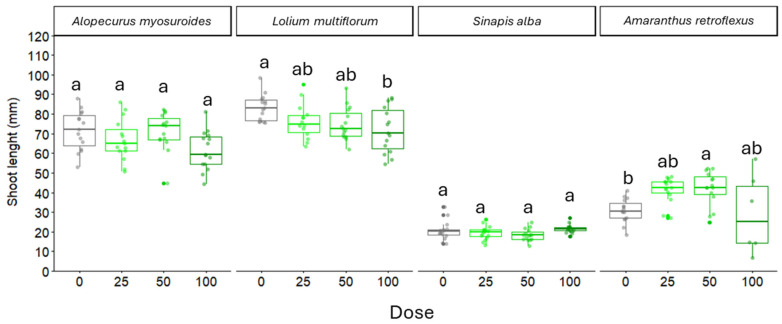
Shoot length of seedlings of *Alopecurus myosuroides*, *Lolium multiflorum*, *Sinapis alba*, *Amaranthus retroflexus*, exposed to water residue (WR) at different concentrations (% of WR: dose 0, dose 25, dose 50, dose 100). Values with the same letter are not significantly different at *p* ≤ 0.05.

In our experiment, shoot length was significantly affected by WR application upon dose (*p* = 0.011), weed species (*p* < 0.0001) and their interaction (*p* = 0.0002). However, the effect on shoot length appeared lower compared to that on roots and did not follow a clear trend related to WR concentration. For *A. myosuroides*, no significant differences in shoot length were observed across WR doses (Figure 3). For *L. multiflorum*, higher WR doses (100) resulted in a significant reduction in shoot length compared to the control (0 dose). In *S. alba*, shoot length remained consistent across all WR doses, showing no significant effect. In contrast, *A. retroflexus* exhibited variability, with significant reductions in shoot length at the highest WR dose (100), while lower doses did not differ significantly from the control (Figure 3).

### 2.3. Chemical Composition of the WR Fraction

**Figure 4:**
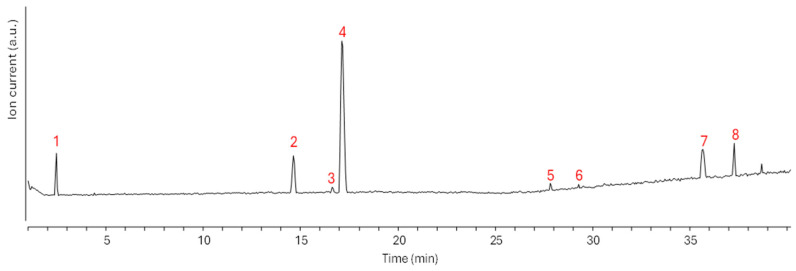
Base peak chromatogram of the WR fraction. Peaks ascribed to identified compounds are numbered according to Table 2.

The HPLC-MS profile of the WR fraction showed a restricted number of peaks. The first peak was ascribed to 3,4-dihydroxyphenyllactic acid (Table 2, Figure 4). This compound is a phenolic acid, a category of low-molecular-weight polyphenols, which can be found in most plant extracts [29]. The most intense peaks were detected in the 14–18 min range. The first of these peaks was ascribed to tuberonic acid. The other two peaks were ascribed to diastereoisomers of gallocatechin, another very common polyphenol of the flavonoid category (Table 2, Figure 4). Other flavonoids were detected at higher retention times in their glycosylated forms: isorhamnetin-3-O-glucoside and hispidulin-7-O-glucoside. The two compounds at the highest retention times were carinol, another polyphenol, and triptolidenol, a diterpenoid (Table 2, Figure 4). Quantitation of detected compounds provided a total extractive concentration of approximately 80 ppm. The highest concentrations were those of tuberonic acid glucoside, gallocatechin, carinol and triptolidenol (Table 2, Figure 4).

## 3. Discussion

The chemical profile of WR, rich in bioactive compounds, such as tuberonic acid, gallocatechin, carinol, and triptolidenol, suggests a potential contribution to the allelopathic activity of WR. Although direct evidence of allelopathic effects for these specific molecules is still limited, our results are consistent with the broader literature, showing that polyphenols and terpenes play important roles in modulating seed germination [30,31]. In general, high concentrations of polyphenols and terpenes tend to reduce the germination rate, acting as growth inhibitors. However, at lower concentrations, these compounds can sometimes even stimulate germination or provide protective effects. The overall impact of these molecules on seed germination is complex, with both positive and negative outcomes [32,33,34]. For example, the effect of gallocatechin, an abundant phenolic compound in WR, on germination varied widely across studies. For instance, some studies reported that a low concentration of gallocatechin and its derivatives support germination, as observed in apple and grape seeds [35,36]. Conversely, its presence in leaf and root extracts has been shown to inhibit seed germination and seedling growth in several weed species, revealing its allelopathic potential [37,38]. Moreover, the crucial role of (±)-catechins on parasitic weed control, e.g., in *Phelipanche ramosa* L., has been recently reported [39].

Notably, in our study, the strong inhibitory effects of WR on *A. retroflexus* germination highlight the sensitivity of this species to phenolic compounds. This aligns with previous findings where catechins ((+)-catechin and (–)-epicatechin), such as those found in walnut leaf extracts, have been identified as significant contributors to the inhibition of *A. retroflexus* germination [40]. In particular, the presence of tuberonic acid in WR may help explain the greater susceptibility of *A. retroflexus* to WR compared to others. Tuberonic acid (12-hydroxyjasmonate), a well-known growth regulator in many plant species [41], belongs to the jasmonic acid family, which is known for enhancing abscisic acid (ABA) activity [42]. ABA plays a key role in suppressing seed germination and post-germinative growth, a process that is particularly significant in species with high seed dormancy, such as *A. retroflexus*.

Previous studies have shown that the effect of allelopathic substances is not necessarily generalizable to all weed species but can be markedly species-specific. For example, Fischer et al. observed that cineole was extremely toxic to *Schizachyrium scoparium* but not to *Leptochloa dubia* [43]. In line with these findings, in our study, the WR caused a marked inhibitory effect on the germination of *A. retroflexus* but showed no significant effects on *L. multiflorum*, *A. myosuroides*, and *S. alba*.

Indeed, other species in our study, such as *A. myosuroides*, showed delayed germination (increased ET50) rather than germination inhibition. This aligns with the findings of Tawaha and Turk, who reported that aqueous extracts of *Brassica nigra* significantly reduced water uptake by *Avena fatua* seeds, thereby delaying germination and subsequent seedling growth [44]. These differences emphasize that allelopathic effects may extend beyond inhibition to also influence germination timing, potentially affecting weed–crop competitive dynamics [45].

Regarding other morphological parameters investigated in this study, root growth inhibition was more pronounced than shoot growth inhibition in most of the weed species tested. This observation aligns with studies on walnut leaf extract, where shoot elongation in *A. retroflexus* and *C. album* was sensibly less affected than root elongation [40]. Similarly, Alipour et al. observed a greater effect of encapsulated rosemary essential oil on the root morphology compared to the aerial part of *A. retroflexus* [46]. Roots, being the first organ to encounter allelochemicals, are particularly sensitive due to their high permeability and direct exposure to compounds in the soil [47,48]. The observed reduction in root length may suggest that cell elongation was also inhibited, as allelopathic agents have been reported to interfere with the functions of gibberellins and indole-3-acetic acid (IAA) [49]. Moreover, similar evidence was also observed in lettuce, where rosemary WR strongly inhibited seedling root growth compared to shoot growth in in vitro experiments [21].

For *A. myosuroides*, root growth showed a dose-dependent reduction under WR treatment with a significant effect already at lower concentrations, whereas *L. multiflorum* showed significant root length inhibition only at the highest concentrations. Similarly, for *A. retroflexus*, a dose-dependent effect was observed, although less evident than in *A. myosuroides*. Such variations in response to the allelopathic substance may be linked to the species-specific growth regulatory effects of allelochemicals, which can also be concentration dependent [50].

The response of *S. alba* to WR was in contrast to what was observed for the other weed species, as root length increased with higher WR concentrations. This observation is supported by similar studies conducted on tomato, where the application of rosemary plant extract increased the seedling root length [51]. For *S. alba*, this could be related to its larger seed reserves, which allow it to sustain root elongation under adverse conditions. This adaptability might represent a survival strategy to escape inhibitory environments by exploring a larger soil volume. This plant response is often observed in cases of drought or nutrient deficiency; in this case, it has also been observed in exposure to potentially allelopathic substances such as WR [52]. Moreover, *S. alba*, in addition to being a widespread weed species in arable and vegetable Mediterranean cropping systems, is also cultivated for seed production, as a fodder crop and as a cover crop, and possesses intrinsic allelopathic and nematocidal traits that may confer enhanced resilience against allelochemicals released by other species; this adaptive capacity could help explain why the inhibitory effect of rosemary extract on *S. alba* was comparatively low [53]. In contrast to what has been observed in this study, Golisz et al. revealed that, although phenolic compounds had a slight effect on the germination of *S. alba* seeds, they inhibited initial root growth [54]. Although the type and magnitude of the negative effects on germination and early growth varied upon weed species, our results confirm the well-established theory indicating that small-seeded species are more sensitive to allelochemicals [55]. For example, the negative effect of WR on initial root growth is inversely correlated with average seed size (0.40, 2.15, 2.20 and 5.53 mg seed^−1^ for *A. retroflexus*, *A. myosuroides*, *L. multiflorum* and *S. alba*, respectively) [50,56]. It can then be hypothesized that regular application of WR-based bioherbicides would cause a shift in weed community composition favoring species with larger seeds, which is a trait typically associated with higher biomass production in the early stages and, hence, higher competitive ability. Therefore, bioherbicide application would not be appropriate as a stand-alone treatment but could be an interesting component of a diversified, integrated weed management strategy, e.g., for agroecological farming [57]. Moreover, it is important to consider whether these allelopathic compounds could have any potential negative effects on the emergence and growth of subsequent crops. In our experiment, the effect of WR was assessed only on weed species as a preliminary trial to evaluate its allelopathic potential. However, the literature shows that the impact of plant extracts can vary widely depending on both the type of aqueous extract and the crop species tested, and no studies to date have specifically investigated WR on crops. However, considering the earlier observation that seed size influences susceptibility to allelochemicals [50], choosing a spring crop with a larger seed size, such as sorghum or maize, may enhance the selective inhibitory action of allelochemicals on weeds while minimizing potential adverse effects on the crop itself.

## 4. Materials and Methods

### 4.1. Plant Material, Extraction Procedure, and Collection of Water Residues (WRs)

WRs were obtained through essential oil extraction of rosemary (cv. Barbeque) grown in a farm located in the Ragusa area (Sicily, Italy). Rosemary was transplanted in a marginal, previously uncultivated area of the farm at a density of 2 plants m^−2^ in rows 25 m long, with an inter-row distance of 2 m. The soil was classified as sandy clay loam, consisting of 50% sand, 20% silt, and 30% clay. Plants were manually harvested at the balsamic stage (flowering) on 15 May 2023, cutting at 10 cm above the ground to simulate mechanical harvesting. Fresh biomass was immediately used for essential oil extraction. More information on the agronomic management of rosemary plants can be found in Formica et al. [2].

The rosemary essential oil extraction process was conducted using a microwave-assisted steam distillation system, based on methods previously reported [6,13,47], with minor modifications. The setup consisted of a commercial stainless-steel steam distillation unit (InHerbam 65L PLUS, InHerbam, Verona, Italy) equipped with a heating boiler, upper and lower grates, a stainless-steel Clevenger-type steam condenser, and a PYREX glass cooling burette. Three coaxial dipole antennas, enclosed in glass tubes, were integrated into the extractor to deliver microwave (MW) energy directly into the fresh plant material [13]. This hybrid system combined MW irradiation with steam produced via conventional gas heating. MW generation was provided by three magnetron oscillators, each with forward and reflected power indicators, capable of delivering up to 1000 W of continuous power at 2.45 GHz.

For each run, 5 L of tap water was placed in the heating boiler, and the lower grate was positioned to isolate the biomass from the steam source. Fresh rosemary (5 kg) was then loaded into the extraction chamber, ensuring full coverage of the MW irradiation zone. The upper grate and Clevenger-type condenser were fitted to seal the unit. After 10 min of conventional gas heating to initiate steam generation, MW irradiation was applied at 600 W total (200 W per magnetron) and maintained along with the steam flow for 60 min under steady-state conditions. Essential oil yield was calculated gravimetrically as the ratio between the mass of oil obtained and the fresh biomass processed. The water residue (WR), collected from beneath the lower grate in the heating boiler, was stored in dark containers at 4 °C until further analysis.

### 4.2. Germination Experiment

The experiment was conducted in vitro, in Petri dishes, to evaluate the effect of WR at different concentrations on the germination and growth parameters of weed seedlings. For this experiment, 4 species of weeds were used, including 2 monocotyledonous, *A. myosuroides* Hudson and *L. multiflorum* Lam., and 2 dicotyledonous, *S. alba* L. and *A. retroflexus* L. These species were selected as they are commonly listed among the most detrimental weeds for arable and vegetable systems in temperate environments. The seeds were purchased from Semances du Puy (Le Puy-en-Velay, France). Before the experiment, the seeds were disinfected by soaking them in a 5% sodium hypochlorite solution for 1 min to eliminate any fungi or spores present on the seed surface [58]. After disinfection, the seeds were washed thoroughly with distilled water to remove any residual chlorine. For each weed species, 60 seeds were placed on moistened filter paper in Petri dishes. Seeds were placed manually in order to maximize the distance between them. WRs were applied upon a decreasing concentration gradient: dose 100 (pure WR), dose 50 (one part of WR and one part of distilled water), dose 25 (one part of WR and three parts of distilled water), dose 0 (only distilled water).

Each combination of weed species and WR concentration was replicated 5 times, for a total of 80 Petri dishes. Each Petri dish (100 mm diameter) was equipped with a paper filter Whatman n°1 (110 mm) soaked with 10 mL of solution. Dishes were sealed to reduce evaporation, and no additional water was required during the tests. The experiment started on 28 February 2024 and ended on 15 March 2024. It was considered complete for each weed species when no additional germination was recorded for three consecutive days following the last observed germination. Petri dishes were incubated at 16 h/20 °C alternating 8 h/16 °C in a dark germination chamber. Petri dishes were checked daily, recording the number of germinated plants and noting the day of germination. A permanent marker was used to mark the germinated seed on the Petri dish lid to track the germination day, facilitating the study of root and shoot elongation dynamics relative to seed germination onset. After the experiment was concluded, the plants were removed from the Petri dishes, and the length of the epicotyl and hypocotyl was measured with an electric caliber. Seeds were considered germinated upon the elongation of a root of at least 1 mm length.

### 4.3. WR Chemical Characterization

The water residue was analyzed using high-performance liquid chromatography coupled with tandem mass spectrometry (HPLC–MS^2^). Measurements were performed on an Agilent Technologies 1200 Infinity HPLC system connected to a 6530 Q-ToF mass spectrometer via a Jet Stream electrospray ionization (ESI) interface. A 3 μL sample volume was injected, with water (A) and acetonitrile (B), each containing 0.14% (*v*/*v*) formic acid, used as mobile phases. Mobile phase flow rate was 0.4 mL/min, and column oven temperature was 40 °C. The following gradients were used: 0–3.75 min. at 100% (A); 3.75–19.50 min. from 100% to 89% (A); 19.50–27.75 min. from 89% to 79% (A); 27.75–44.25 min. from 79% to 60% (A); 44.25–50.25 min. from 60% to 39% (A); 50.25–51 min. from 39% to 0% (A); 51.00–52.50 at 0% (A). Re-equilibration time was 12 min. For the ESI interface, the operating parameters were as follows: drying gas (N_2_, 98% purity) at 10 L min^−1^ and 350 °C; sheath gas (N_2_, 98% purity) at 11 L min^−1^ and 375 °C; capillary voltage set to 4.5 kV; nebulizer gas pressure at 35 psig; and collision-induced dissociation (CID) voltage of 20 V. High-resolution MS and MS^2^ data were acquired over an *m*/*z* range of 100–1000, using negative ion mode for all analyses. The mass spectrometer was calibrated daily with Agilent tuning mix HP0321 (Agilent Technologies, Santa Clara, CA, USA) dissolved in acetonitrile. Data processing was performed with MassHunter Qualitative Analysis software (version B.04.00). Compounds were identified based on their MS and MSMS spectra, by comparison with available literature references [30,59,60]. Quantitative analysis was performed to assess the concentrations of the main compounds extracted in the WR. Concentrations were calculated as catechin equivalents by building a calibration curve using a reference catechin (≥96%, Sigma-Aldrich (Darmstadt, Germany), calibration range 0.5–20 ppm, regression R^2^ = 0.998).

### 4.4. Statistical Analysis

The germination data were fitted to a Weibull distribution function to study germination over time at different concentrations of WR for each weed species separately, using a dose–response curve model. This was done to obtain estimates of three main parameters of seed germination: the upper limit of germination (Max), the slope at the inflection point (b), and the time taken to achieve 50% germination (ET50). Each parameter was calculated for each set of water extraction doses. The analysis was performed using the drc package for R [61]. Variance–covariance matrix (*p* ≤ 0.05) was used to identify significant differences between parameter values estimated for the different extraction water concentration using sandwich package [62].

Root and shoot lengths were analyzed using a Linear Mixed Model (LMM) implemented in the lme4 package for R [63]. Fixed factors included weed species (4 levels: *A. myosuroides*, *L. multiflorum*, *S. alba*, *A. retroflexus*), WR doses (4 levels: D0, D25, D50, D100), and their interaction, while random effects consisted of individual plants nested within replicates (blocks). Based on the analysis of variance results, Sidak post hoc tests were conducted to separate means for significant explanatory variables (*p* < 0.05), using the R/emmeans package [64]. Residuals were assessed for normality and homogeneity of variance using the Kolmogorov–Smirnov and Levene tests, respectively, with the R/DHARMa package [65]. To explore root length development over time (days after treatment) and WR concentration, linear contrasts were employed to compare the trends (slopes) of each interaction level to zero. Differences in slopes were then tested for statistical significance (*p* < 0.05) using the R/emmeans package [64].

## 5. Conclusions

Overall, the species-specific responses observed in our study highlight the potential of WR as a selective natural herbicide. The high sensitivity of *A. retroflexus* highlights the potential use of WR for the management of detrimental weeds. Moreover, the observed ability of WR to delay germination and seedling development in weeds can represent a valuable strategy to give crops a competitive advantage over weed development. However, while many studies have shown no significant adverse effects of WR on the germination of major crops, fine-tuning its application to enhance selectivity and minimize potential risks to non-target species remains a key challenge. The inhibitory properties of WR represent a valuable solution for valorizing this product, transforming what is normally considered waste of the essential oil extraction process into a sustainable tool for weed management. By leveraging the allelopathic WR properties, its integration into integrated weed management (IWM) strategies could reduce dependence on synthetic herbicides, contributing to more sustainable farming systems.

## Figures and Tables

**Table 1:** Parameters related to the germination curves of the target weed species *Alopecurus myosusoides*, *Lolium multiflorum*, *Sinapis alba*, *Amaranthus retroflexus* in response to different concentrations of water residue (WR). The parameters include slope at inflection point (b), upper limit of germination (Max), and time to reach 50% of germination (ET50). Different letters indicate significant differences within each parameter and independently for each weed species. Abbreviations: DAT, day after treatment.

Weed Species	Dose	Slope	Upper Limit	ET50 (DAT)
*Alopecurus* *myosuroides*	0	−4.53 (0.28) c	0.93 (0.01) a	3.84 (0.06) d
	25	−3.94 (0.24) bc	0.96 (0.01) a	4.21 (0.07) bc
	50	−3.73 (0.23) ab	0.95 (0.01) a	4.34 (0.08) b
	100	−3.24 (0.20) a	0.96 (0.01) a	4.99 (0.11) a
*Lolium* *multiflorum*	0	−3.97 (0.25) a	0.88 (0.01) a	2.21 (0.04) c
	25	−3.90 (0.24) a	0.86 (0.01) a	2.34 (0.04) b
	50	−3.77 (0.22) a	0.90 (0.01) a	2.41 (0.04) ab
	100	−3.46 (0.20) a	0.91 (0.01) a	2.47 (0.05) a
*Sinapis alba*	0	−0.84 (0.16) c	1.00 (0.06) a	0.74 (0.07) d
	25	−1.35 (0.15) b	1.01 (0.02) a	0.97 (0.05) c
	50	−1.58 (0.15) ab	1.02 (0.01) a	1.12 (0.05) b
	100	−1.89 (0.14) a	0.99 (0.01) a	1.29 (0.04) a
*Amaranthus* *retroflexus*	0	−4.25 (0.37) b	0.48 (0.02) a	1.79 (0.05) cd
	25	−3.45 (0.33) ab	0.30 (0.02) b	2.69 (0.09) a
	50	−3.60 (0.46) ab	0.17 (0.02) c	2.60 (0.11) ab
	100	−1.51 (0.46) a	0.04 (0.01) d	2.19 (0.37) abcd

**Table 2:** List of compounds identified in the WR fraction by HPLC-MS analysis. For each compound, the table shows the brute formula, molecular weight (MW), pseudomolecular ion (PMI), and main signals in the MS spectra. The last column shows the concentration in parts per million equivalents of catechin.

#	Compound	Formula	MW (amu)	PMI	MSMS Signals	C (ppm)
1	3,4-Dihydroxyphenyllactic acid	C_9_H_10_O_5_	198.2	197.05	179, 135, 123	7.3
2	Tuberonic acid glucoside	C_18_H_28_O_9_	388.4	387.17	207, 163, 119	33.4
3	Gallocatechin 1	C_15_H_14_O_7_	306.3	305.07	225, 97	2.4
4	Gallocatechin 2	C_15_H_14_O_7_	306.3	305.07	225, 97	12.1
5	Isorhamnetin 3-O-glucoside	C_22_H_22_O_11_	478.4	477.10	315, 299	0.2
6	Hispidulin 7-O-glucoside	C_22_H_22_O_11_	462.4	461.11	283	2.7
7	Carinol	C_20_H_26_O_7_	378.4	377.16	359, 315, 289, 211, 165	11.5
8	Triptolidenol	C_20_H_24_O_7_	376.4	375.16	331, 313, 303, 287, 275, 259	10.7

## Data Availability

Data available upon request.

## Abbreviations

The following abbreviations are used in this manuscript:

WRs	Water Residues
